# Rare biochemical & genetic conditions: clues for broader mechanistic insights

**DOI:** 10.1007/s00018-025-05652-6

**Published:** 2025-04-10

**Authors:** Alexios-Fotios A. Mentis, Maria Dalamaga

**Affiliations:** Present Address: Department of Biological Chemistry, Medical School, National and Kapodistrian University of Athens, Athens, Greece

**Keywords:** Metabolism, Orphan disorders, Genetics, Inborn errors of metabolism, Cancer, Chemistry, Rare disorders

## Abstract

Rare disorders often represent a molecular deviation from hi-fidelity genomic integrity networks and are often perceived as too difficult or unimportant for further mechanistic studies. Here, we synthesize evidence demonstrating how valuable knowledge of biochemical pathways related to rare disorders can be for biomedicine. To this end, we describe several rare congenital lipid, protein, organic acid, and glycan metabolism disorders and discuss how rare phenotypes (such as “*extreme responders*”) and case reports (such as the lenalidomide cases) have provided clues for drug discovery or repurposing. We also discuss how rare disorders such as Gaucher disease and ultra-rare genetic syndromes can provide insights into cancer and mTOR-driven metabolism, respectively. Our discussion highlights the continued value of biochemical pathways and studies in understanding human pathophysiology and drug discovery even in the genomics era.

## Introduction

The integrity of human genomic and metabolic processes is maintained through a complex set of biochemical pathways, from DNA damage repair mechanisms [1] to elaborate molecular pathways involving histone modifications and genetic recombination [2]. There is increasing evidence that such recombination events, such as crossovers, are not stochastic but are orchestrated to minimize the frequency of de novo mutations in functional proteins, thereby reducing the incidence of genetic disorders [3]. However, there exist rare variants, operationally defined as “*single nucleotide polymorphisms with a minor allele frequency of less than 0.01*” [4], which, despite their extremely low prevalence, often have profound impacts on human disease-associated phenotypes. These rare variants—in most cases—are either typical variants for rare genetic and biochemical disorders or rare variants of common disorders [5].

**Fig. 1:**
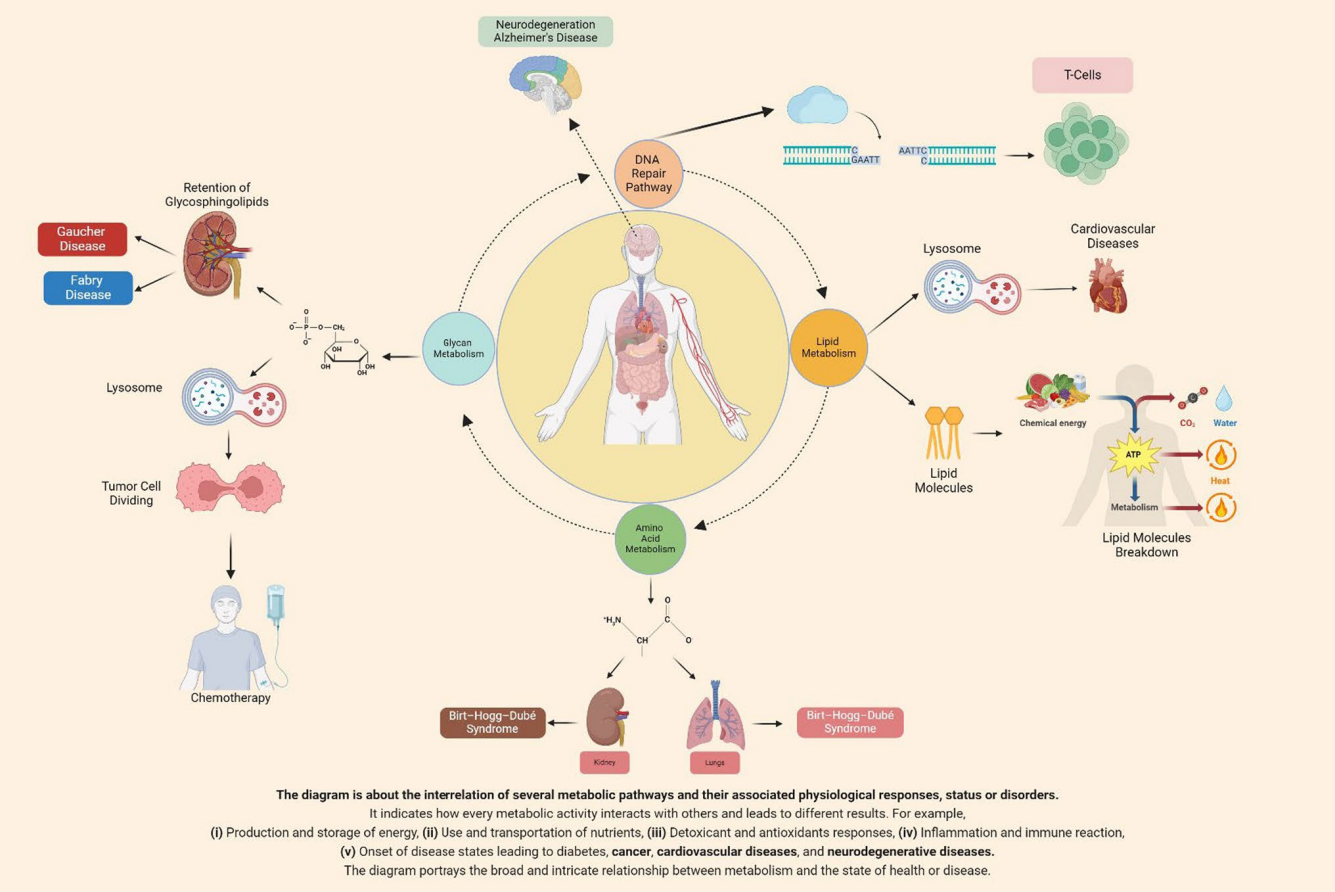
Graphical abstract of the rare biochemical and genetic conditions and pathways that can provide broader insights to human pathophysiology

**Fig. 2:**
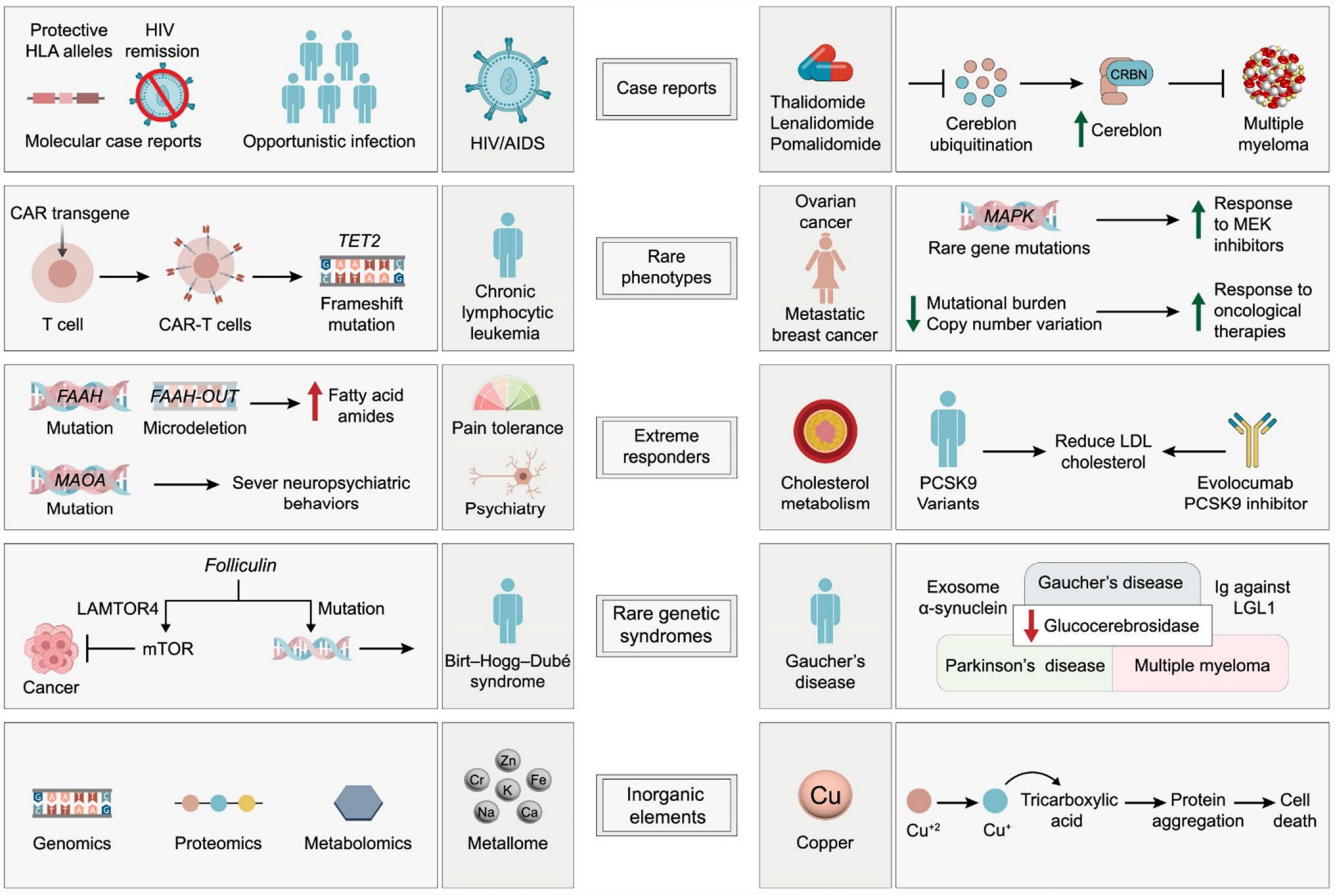
Illustrative examples of case reports, rare phenotypes, extreme responders, rare genetic syndromes, and inorganic elements that provide broader mechanistic insights into different diseases

Though rare, the above disorders are a feature of human life and are now more effectively diagnosed than in the past [6]. In clinical practice, these disorders tend to be appreciated only within the context of rare phenotypes and regarded as of only limited value for understanding complex human traits and polygenic disorders, usually treated and managed by specialists (such as clinical geneticists, metabolic genetics specialists, etc.). Here, we posit that studying rare genotypes and diseases—whether genetic or biochemical—can provide significant and impactful mechanistic insights into broader aspects of human pathophysiology, including common, frequently occurring disorders. This is true even in an era demanding solution to major public health issues through high-quality evidence-based medicine (i.e., systematic reviews and clinical trials). To support our thesis, we provide curated, compelling evidence coupled with brief mechanistic insights from the current literature (Figs. 1 and 2). In so doing, we argue that case reports have added value to the broader field of medicine; we provide compelling evidence on how rare genetic syndromes and extreme responders can provide broader clues both for mechanistic understanding and drug development; we illustrate how phenotypically distinct diseases can share common but neglected biochemical backgrounds; and we highlight the importance of inorganic (as opposed to organic) pathways in human biochemistry.

## Case reports: a valuable tool in clinical, molecular, and biochemical medicine

Since the landmark publication by Guyatt et al. [7], clinical medicine has increasingly relied on evidence-based approaches, such as systematic reviews and meta-analyses, rather than expert opinion. However, case reports and case series have consistently demonstrated the value of case descriptions to clinical medicine, as evidenced by the development of the CARE guidelines to standardize case report presentations [8] (for further discussion on the value of medical case reports, see: [9]).

Two indicative (but by no means exhaustive) examples of the value of case reports are the history of HIV discovery and the drug repurposing of thalidomide and its analogs. The initial descriptions of HIV/AIDS, based on five cases of unprecedented opportunistic infection, exemplify the crucial role played by case reports in raising awareness of important diseases [10]. More recently, equally important “*molecular case reports*” of patients with AIDS who, on the one hand, presented with HIV remission in the presence of clonally expanded, replication-competent (and, in no case, defective) HIV viral strains and who, on the other, had no protective HLA alleles, provide major clues about the molecular determinants of HIV “*cure*” [11].

Similarly, the teratogenic effects of thalidomide, initially used to treat morning sickness in pregnant women, led to the repurposing of thalidomide analogues like lenalidomide and pomalidomide as immune-modulating drugs in patients with multiple myeloma [12] based on a mechanism whose elucidation was both surprising and complex (Box 1). These examples illustrate how case reports of rare phenotypes and congenital malformations have driven drug development for rare diseases.

Box 1. Mechanism of action of thalidomide Thalidomide represents a useful case study on (a) how a drug can be repurposed from one disease to a phenotypically distinct disease; and (b) the challenges in establishing precise mechanism. Mechanistically, thalidomide was identified as a protein that binds to cereblon, inhibiting its auto-ubiquitination [50]. Deletion of the cereblon gene in zebrafish caused limb abnormalities like those induced by thalidomide. Cereblon, an E3 ubiquitin ligase, binds to DDB1, Roc1, and Cullin-4A, leading to protein degradation. Thalidomide disrupts this complex [51]. However, two clinical observations led researchers to reconsider thalidomide analogues: (a) higher cereblon levels in multiple myeloma cells correlated with better responses to immune-modulating drugs, and (b) drug-resistant myeloma cells had lower cereblon levels [52]. The above findings, coupled with experimental evidence, suggest that thalidomide analogs alter cereblon’s substrate specificity rather than antagonizing cereblon. In so doing, lenalidomide was found to induce ubiquitination and proteasomal degradation of Ikaros and Aiolos, two zinc finger transcription factors that are bound by cereblon and that are critical for plasma and B cells [50, 52]. The lesson learned above based on the otherwise well-discussed case of thalidomide is the need for being meticulously sophisticated when designing experimental studies to elucidate molecular, genetic, and biochemical mechanisms of action for both understanding diseases and developing drugs.

## Rare phenotypes and genetic syndromes: molecular clues to broader mechanisms?

### Rare phenotypes and extreme responders

The concept of “*rare phenotypes*”, often referred to as “extreme responders”, has attracted attention over the last few decades, primarily in the context of cancer therapeutics. For example, ovarian cancer patients with rare *MAPK* gene mutations tend to respond more favorably to MEK inhibitors [13]. Similarly, patients with metastatic breast cancer with a genetic profile characterized by low mutational burden and fewer copy number variations exhibit significantly higher response rates to oncological therapies [14]. In a similar context, a particularly compelling case involves a patient with chronic lymphocytic leukemia who achieved complete remission following CAR-T cell therapy targeting the CD-19 protein. Molecular investigation revealed that nearly all CAR-T cells originated from a unique clone, where the insertion of the CAR transgene via viral delivery caused a frameshift mutation in the DNA methylation gene *TET2* [15]. These examples illustrate how rare genetic mutations can significantly alter the clinical management of challenging diseases, paving the way for generalizable research and therapeutics.

The study of extreme responders has also informed therapeutic developments beyond cancer. For instance, individuals with variants in the proprotein convertase subtilisin/kexin type 9 (*PCSK9*) gene, which reduce the risk of cardiovascular disease by half, inspired the development of evolocumab, a monoclonal antibody targeting the PCSK9 protein, now tested in both adults and children [16, 17]. Notably, *PCSK9* variants affect low-density lipoprotein cholesterol levels, with effects comparable to and additive with those of certain *HMGCR* variants, which influence statin therapy responsiveness [18].

A more recent molecular case report describes an extreme responder of pain insensitivity characterized by extraordinary pain tolerance, including no requirement for postoperative analgesia, as well as rapid and painless healing from burns and other injuries [19]. The phenotype of pain insensitivity was linked to a combination of mutations in the fatty-acid amide hydrolase (*FAAH*) gene (which causes the hydrolysis of, and, in turn, inactivates endogenous bioactive fatty acid amides, such anandamide and oleamide), and a microdeletion in the *FAAH-OUT* pseudogene; of note, the latter functions as a large non-coding RNA expressed in dorsal root ganglia [19]. In addition, levels of fatty acid amides were significantly higher in these patients compared to normal controls, thus mimicking endocannabinoid signalling [19]. Therefore, extreme genetic and biochemical phenotypes can provide mechanistic clues, even for typically unrelated aspects of human physiology, such as cholesterol metabolism and pain tolerance [19].

Furthermore, rare phenotypes and biochemical conditions can provide valuable insights into neuropsychiatric and psychiatric traits, though they require much more scrutiny due to the complexity of the human brain and mind. It is worth noting that, under certain circumstances, patients with rare mutations in genes encoding metabolic enzymes that affect neurotransmitters, such as the monoamine oxidase A (*MAOA*) gene, can exhibit severe neuropsychiatric behaviors, thus linking biochemistry to psychiatry. However, how generalizable and instructive these observations are for other similar traits still needs to be explored [20].

### Rare genetic syndromes: a source of valuable mechanistic insights

Besides rare phenotypes and extreme responders, rare genetic syndromes hold particular value for understanding human pathophysiology. For instance, Gaucher’s disease, a rare metabolic disorder caused by mutations in the glucocerebrosidase gene, which breaks down glucocerebroside, has unexpectedly provided significant insights into blood cancer [21]. In patients with Gaucher’s disease, the accumulation of lyso-glucosylceramide (LGL1) leads to the development of clonal immunoglobulins reactive against LGL1, increasing the risk of monoclonal gammopathies [22]. This finding also revealed that one-third of sporadic multiple myeloma cases may involve clonal immunoglobulins against LGL1 or lysophosphatidylcholine, highlighting new antigens linked to oncogenesis [22]. Given this association, patients with monoclonal gammopathies such as multiple myeloma are now screened for Gaucher’s disease [23]. Of note, the broader significance of Gaucher’s disease is further emphasized by its association with Parkinson’s disease (PD), where the activity levels of glucocerebrosidase (defective in Gaucher’s disease) are linked to two majors features of Parkinson’s disease, exosome production and secretion of pathological α-synuclein [24]. Likewise, there are several new lines of evidence linking glucose-6-phosphate dehydrogenase (G6PD) deficiency with PD: (i) clinically relevant variants of the G6PD associated with G6PD deficiency are associated with a diagnosis of PD and its pathology; (ii) accumulation α-synuclein micro-aggregates affects G6PD-related pentose phosphate pathways, leading to reductions in nicotinamide adenine dinucleotide phosphate and glutathione levels and, in turn, dopamine oxidation, whose impairment is traditionally associated with PD; and, (iii) conversely, recovery of nicotinamide adenine dinucleotide phosphate and glutathione levels reverses dopamine oxidation and PD-related pathology [25]. Therefore, these are examples of rare biochemical disorders that can be associated with diseases of major clinical and public health importance.

Another indicative example is Birt–Hogg–Dubé syndrome, a genetic disorder caused by mutations in the folliculin gene, which causes kidney and skin tumors. Recent studies have linked mTOR signaling to lysosomal organelle formation, partly mediated by interactions between the folliculin and LAMTOR4 proteins [26, 27]. Conversely, folliculin influences mTOR signaling both directly and indirectly, and its interaction with LAMTOR4 can induce synthetic lethality in cancer cells, suggesting a potential new therapeutic approach [27]. In this way, a rare syndrome offers valuable insights into both metabolism (exemplified through the key mTOR pathway) and cancer pathogenesis.

## How can molecular pathways unify distinct diseases?

The discussion above highlights the importance of rare but distinct features (phenotypes, syndromes, or molecular responses) to human pathophysiology. However, distinct diseases like cancer and inborn errors of metabolism can share common and/or overlapping molecular pathways. For example, the rare biochemical disorder D-2-hydroxyglutaric aciduria type 2, which is caused by germline mutations in the isocitrate dehydrogenase 2 (*IDH2*) gene and which causes progressive brain damage, is treated with enasidenib, a drug that has been approved to treat a blood cancer, namely acute myeloid leukemia caused by somatic mutations in the *IDH2* gene [28, 29], Similarly, lonafarnib, a farnesyltransferase inhibitor with antineoplastic properties, also reduces Parkinson-related tau pathology through a lysosome-activating mechanism involving the Rhes protein [30]. These approaches show how drugs can be repurposed based on shared mechanisms between apparently different disorders.

Additionally, biochemical substances previously considered byproducts of cellular metabolism, such as urea and lactate, have recently attracted considerable attention due to their mechanistic value. First, urea, a byproduct of nitrogen metabolism, has been reported as a cancer biomarker by increasing pyrimidine-to-purine conversion. The latter increases the number of DNA and RNA transversion mutations and, as such, increases the rates of hydrophobic tumor neoantigens responsive to immunotherapy [31]. Even if the precise impact of several variants remains unclear, enzymes that are part of the urea cycle (e.g., ornithine transcarbamylase) appear to provide metabolic clues about cancer survival and development, an area of potential future research interest [32, 33]. Likewise, phenylalanine substitutions at the codon reassignment level enhance the repertoire of cell surface antigens under conditions of tryptophan depletion [34]. Third, lactate has recently been shown to be involved in DNA repair (namely, homologous recombination-mediated pathways) through lactylation of the Nijmegen breakage syndrome 1 (*NBS1*) protein. In turn, lactate is associated with resistance to DNA damage-causing chemotherapy which can, however, be reversed by stiripentol, an anti-epileptic drug with lactate dehydrogenase inhibitory activity [35].

## Specific metabolic pathways and their implications for disease

Shifting the focus from general biochemical pathways and phenotypes, specific lipid, protein, and glycan metabolism pathways can also provide insights into pathophysiological processes.

### Lipid metabolism: triacylglycerol formation and its broader insights

Lipid metabolism-related biochemistry may be regarded as completely understood, leaving little room for novel discoveries. However, recent studies challenge the traditional view of a single biochemical pathway for triacylglycerol formation via diacylglycerol O-acyltransferase–mediated synthesis from coenzyme A-conjugated fatty acids [36]. Instead, DIESL, usually thought of as an acyltransferase of unknown function, was identified as a novel triacylglycerol synthase regulated at the endoplasmic reticulum by TMX1 and activated in response to extracellular lipid scarcity. This mechanism may have implications for neurodegeneration and cancer [36]. These observations underscore that, even after more than half a century since the initial description of triacylglycerol formation, there are still valuable lessons to be learned from human biochemistry.

### Amino acids: the far-reaching implications of tetrahydrobiopterin metabolism

Among the many different co-factors, tetrahydrobiopterin represents a key example of “transferable” mechanistic insights from amino acid metabolism (and of aminoacidopathies) to other diseases categories. As an enzymatic cofactor for phenylalanine hydroxylase, tetrahydrobiopterin deficiency has been identified in patients with elevated phenylalanine levels not linked to phenylalanine hydroxylase deficiency but with phenylketonuria-related features [37]. Remarkably, recent studies have shown that tetrahydrobiopterin interacts not only with enzymes but also with the non-coding RNA *HULC*, which in turn affects phenylalanine hydroxylase gene expression [38]. Also, tetrahydrobiopterin deficiency is implicated in a lysosomal storage disorder, Fabry disease, where substrate reduction therapy has restored tetrahydrobiopterin levels to improve heart and kidney pathology [39]. Of note, tetrahydrobiopterin’s role has expanded beyond inborn errors of metabolism, with evidence showing that it regulates T cell proliferation and antigen presentation. In vivo, tetrahydrobiopterin shows antitumor activity by increasing effector T cells, while inhibition of its formation increases allergic and autoimmune reactions [40]. These findings reveal a much broader role for a single co-factor (tetrahydrobiopterin); thus, similar exploration of other enzymatic cofactors is also warranted.

### Congenital disorders of glycosylation

The metabolism of glycans is perhaps less well studied than that of proteins or lipids. From a clinical perspective, most patients with congenital disorders of glycosylation are identified through neonatal screening based on analyzing serum transferrin glycosylation [41]. Nonetheless, recent findings on a congenital disorder of glycosylation caused by *DHRSX* gene mutations have shifted our understanding of dolichol metabolism, a key carrier for oligosaccharides during N-glycosylation [42]. This disorder exhibits pseudoautosomal recessive inheritance, linked to the evasion of X-inactivation and seen in individuals with sex chromosome aneuploidies [42]. In so doing, the traditional view of dolichol production as a one-step process mediated by SRD5A3 has been challenged. SRD5A3 is now understood to mediate only the second step of a three-step process, with the other two reactions catalyzed by DHRSX, which enhances the reduction of carbon–carbon double bonds [42]. DHRSX also has dual specificity for NAD and NADP, enabling it to catalyze either dehydrogenation or reduction [42]. The field of inborn errors of metabolism is consistently evolving, in part because increased understanding of fundamental biochemical principles is fueling mechanistic discoveries on human biochemical disorders.

## Inorganic biochemical components: a role not to be neglected

Organic components, such as those mentioned above, are not the only biochemical links to human pathophysiology; inorganic biochemical components are also crucial but relatively neglected. Historically, trace elements, despite being indispensable components of various enzymatic processes, have not been extensively studied regarding their biological and metabolic impacts. However, recent research has renewed interest in this area. For instance: (a) copper is implicated in cell death processes by binding to tricarboxylic acid-related proteins, leading to protein aggregation-induced proteotoxic stress [43]; and (b) the “*metallome*” has now been recognized as an age-dependent major determinant of metabolic health and is considered a previously overlooked omics layer compared to genomics, proteomics, and metabolomics [44]. As a result, future *-omics* studies should include the effects of inorganic compounds on human pathophysiology.

## How can biochemical pathways contribute to drug development?

Genetic and biochemical screening has revealed unprecedented associations between fundamental biochemical pathways—even at the level of amino acids—and significant genetic effects (for a major example, see Box 2), such as those involved in neurodegeneration or cancer. For example, glutaminyl-peptide cyclotransferase is an enzyme that catalyzes the conversion of N-terminal glutamine and glutamate residues into N-terminal pyroglutamate through cyclization [45]. Notably, because glutaminyl cyclase mediates the formation of a pathological hallmark of Alzheimer’s disease, amyloid-beta, the inhibitor varoglutamstat was developed and even tested in phase II clinical trials, although outcomes were disappointing [46]. However, despite the lack of success in Alzheimer’s disease, glutaminyl-peptide cyclotransferase remains a promising target for anti-cancer drugs, given the multidimensionality of shared biochemical pathways. Interestingly, a glutaminyl-peptide cyclotransferase-like protein appears to influence the binding of signal regulatory protein α (SIRPα) to the CD47 protein, which in turn activates the *MYC* oncogene and generates the ‘don’t eat me’ signal that protects cancer cells from myeloid cells [47].

Box 2. Natural and modified amino acids: tiny molecules with major mechanistic and therapeutic potential! To appreciate the profound impact of essential biochemical elements, such as amino acids, on human pathophysiology, we provide two examples. First, N-acetyl-L-leucine, a synthetic version of the essential amino acid leucine, has demonstrated efficacy in the treatment of three major lysosomal disorders caused by enzymatic defects: Niemann-Pick disease type C, Tay-Sachs disease, and Sandhoff disease [53, 54]. Second, diseases attributed to the dysregulated metabolism of a single amino-acid—namely serine—due to mutations in subunits of serine palmitoyltransferase (or “serineopathies”) result in the accumulation of abnormal deoxysphingolipids instead of the normal production of sphingolipids, ultimately leading to retinopathies and peripheral neuropathies [55]. These examples showcase how even a single molecule can affect the entire organism.

## Conclusions: future avenues and challenges

Genomic medicine undeniably embodies the 21st-century equivalent of the zeitgeist that medical biochemistry represented in the twentieth century, with some overlap between the two [48]. This trend is epitomized in the field of RNA-based medicine, which includes antisense, RNAi-based, and mRNA-based therapeutics, delivered either ex vivo or in vivo and applicable to both eukaryotic and prokaryotic organisms [49]. However, our perspective highlights the still vital and necessary role of biochemical case reports, extreme respondents and phenotypes, and inborn errors of metabolism in understanding human pathophysiology. In so doing, these insights from rare and biochemical diseases provide interdisciplinary relevance to fields like genomics, pharmacology (e.g., drug discovery or repurposing), and even public health (e.g., public health screening for biochemical disorders in broader populations). They can also be a call-for-action to clinical practitioners so that they refine their medical “clairvoyance” for rare phenotypes, which can then serve as springboard for broader biomedical discoveries. Biomedical and health policymakers must, in turn, further fund rare disorders. Importantly, though, the zeal for scientific discoveries should not overwhelm the need for appropriate bioethical standards: the need for patient consent, maintenance of data privacy, and the right of patients to beneficence from the research findings.

## Data Availability

Not applicable.
